# Artificial intelligence for predicting five-year survival in stage IV metastatic breast cancer patients: A focus on sarcopenia and other host factors

**DOI:** 10.3389/fphys.2022.977189

**Published:** 2022-09-27

**Authors:** Woocheol Jang, Changwon Jeong, KyungA Kwon, Tae In Yoon, Onvox Yi, Kyung Won Kim, Seoung-Oh Yang, Jinseok Lee

**Affiliations:** ^1^ Department of Biomedical Engineering, Kyung Hee University, Yongin, South Korea; ^2^ Department of Electronics and Information Convergence Engineering, Kyung Hee University, Yongin, South Korea; ^3^ Medical Convergence Research Center, Smart Business Team in Information Management Office, Wonkwang University Hospital, Wonkwang University, Iksan, South Korea; ^4^ Department of Nuclear Medicine, Dongnam Institute of Radiological and Medical Sciences, Busan, South Korea; ^5^ Department of Hemato-Oncology, Dongnam Institute of Radiological and Medical Sciences, Busan, South Korea; ^6^ Department of Surgery, Dongnam Institute of Radiological and Medical Sciences, Busan, South Korea; ^7^ The Department of Radiology and Research Institute of Radiology, Asan Image Metrics, Clinical Trial Center, Asan Medical Center, University of Ulsan College of Medicine, Seoul, Korea

**Keywords:** breast cancer, artificial intelligence, feature importance, sarcopenia, five-year survival

## Abstract

We developed an artificial intelligence (AI) model that can predict five-year survival in patients with stage IV metastatic breast cancer, mainly based on host factors and sarcopenia. From a prospectively built breast cancer registry, a total of 210 metastatic breast cancer patients were selected in a consecutive manner using inclusion/exclusion criteria. The patients’ data were divided into two categories: a group that survived for more than 5 years and a group that did not survive for 5 years. For the AI model input, 11 features were considered, including age, body mass index, skeletal muscle area (SMA), height-relative SMA (H-SMI), height square-relative SMA (H^2^-SMA), weight-relative SMA (W-SMA), muscle mass, anticancer chemotherapy, radiation therapy, and comorbid diseases such as hypertension and mellitus. For the feature importance analysis, we compared classifiers using six different machine learning algorithms and found that extreme gradient boosting (XGBoost) provided the best accuracy. Subsequently, we performed the feature importance analysis based on XGBoost and proposed a 4-layer deep neural network, which considered the top 10 ranked features. Our proposed 4-layer deep neural network provided high sensitivity (75.00%), specificity (78.94%), accuracy (78.57%), balanced accuracy (76.97%), and an area under receiver operating characteristics of 0.90. We generated a web application for anyone to easily access and use this AI model to predict five-year survival. We expect this web application to be helpful for patients to understand the importance of host factors and sarcopenia and achieve survival gain.

## Introduction

Breast cancer (BC) is the most common malignant cancer in women. With advances in diagnosis and treatment, there have been significant improvements in breast cancer outcomes (DeSantis et al., 2019). The overall five-year survival rate is approximately 90 percent for all stages. However, stage IV metastatic breast cancer has remained a dismal disease with a poor prognosis. Indeed, according to the American Cancer Society, the five-year survival rate after diagnosis for people with stage IV breast cancer is 28 percent (Miller et al., 2019).

Therefore, it is essential to more precisely identify metastatic breast cancer patients at a high risk of mortality so that we can tailor the therapy for such patients. Several prognostic factors have been identified, which can be categorized as follows: (DeSantis et al., 2019) tumor factors such as size, grade, stage, lymph node involvement, hormone receptor status, and molecular subtypes; (Miller et al., 2019) host factors such as age, ethnicity, socioeconomic status, and sarcopenia; and (Ho-Huynh et al., 2019) treatment factors such as type of chemotherapy (Bonotto et al., 2014; Gobbini et al., 2018; Ho-Huynh et al., 2019). The predictive performance of treatment factors varies due to the continuous development of new drugs such as targeted agents and immunotherapeutic agents. Therefore, host factors have gained huge emphasis because of the increase in the number of long-term survivors.

Among host factors, sarcopenia is gaining emphasis as a prognostic factor of mortality in breast cancer patients (Villasenor et al., 2012; Shachar et al., 2017; Caan et al., 2018; Song et al., 2018; Weinberg et al., 2018). Sarcopenia refers to the decreased skeletal muscle mass and strength/function in cancer patients and is closely related to quality of life, physical disability, and mortality (Lee et al., 2019). As cancer patients live longer, sarcopenia might become more influential for survival. In metastatic breast cancer patients, sarcopenia can be evaluated by measuring skeletal muscle mass on computed tomography (CT) scans. Currently, there is sparse evidence to evaluate the prognostic value of sarcopenia in metastatic breast cancer (Song et al., 2018). Recently, a few studies presented machine learning models for the prediction of survival or mortality (Abdikenov et al., 2019; Simsek et al., 2020; Haque et al., 2022; Lin et al., 2022). However, the studies did not consider body composition measures as input features. In this study, we considered the body composition measures and presented AI model to predict the mortality for the patients with stage IV metastatic breast cancer.

In the era of personal communication devices, patients and doctors are interested in using a simple application to predict the survival of cancer patients. In general, five-year survival is regarded as an important milestone for cancer patients. Thus, an easily accessible tool for the prediction of five-year survival would be beneficial to cancer patients as well as doctors. To date, there has been limited use of prediction tools in clinical practice because they require a large number of input variables or sophisticated statistical calculation methods.

Therefore, we aim to evaluate the prognostic value of sarcopenia measured on CT and develop an artificial intelligence application, such as a public website, using a small number of input variables to easily predict five-year survival in metastatic breast cancer patients.

The main contributions of this study can be summarized as follows. First, we collected 226 patients with stage IV metastatic breast cancer on the basis of a prospectively built breast cancer registry. Second, we considered body composition measures such as skeletal muscle area and other related values for our AI model inputs. Third, we performed the feature importance analysis indicating the order of importance among the features. Last, we presented cross-validation results and further validated our proposed model from an isolated testing dataset.

The rest of the paper is organized as follows. Section 2 describes data characteristics, preprocessing and our proposed AI model. In Section 3, the results of ranked feature importance, cross-validation results, testing data results and deployed web application are presented. Finally, in Section 4, we conclude the paper with summary and discuss the future work.

## Methods

This study was approved by the institutional review board of Dongnam Institute of Radiological Medical Science, Busan, Korea (IRB No. D-2104-001-002). Informed consent was waived. All methods were performed in accordance with the relevant guidelines and regulations.

### Patient data for the AI model

The AI model for five-year survival prediction after the date of diagnosis of stage IV breast cancer was constructed on the basis of a prospectively built breast cancer registry at Dongnam Institute of Radiology and Medical Sciences (DIRMS) between 2010 and 2020. In this study, we enrolled 226 patients, each of whom had 11 medical records, to predict their five-year survival. Among a total of 226 patients, we excluded those surviving patients who were diagnosed less than 5 years from the study period (n = 16). Then, data from DIRMS for a total of 210 patients were used for training and testing our AI model.

Each patient record includes 17 variables such as the patient’s ID, age, weight, height, body mass index (BMI), skeletal muscle area (SMA), height-relative SMA (H-SMI), height square-relative SMA (H^2^-SMA), weight-relative SMA (W-SMA), muscle mass, anticancer chemotherapy, radiation therapy, comorbid diseases (hypertension and mellitus), date of diagnosis, date of death, and survival period. Table 1 summarizes the 17 variables for each patient.

**TABLE 1:** Variables from patients’ data.

	Data	Type	Description
1	ID	Number	Anonymous
2	Age	Number	age
3	Weight	Number	Weight (unit = kg)
4	Height	Number	Height (unit = cm)
5	BMI	Number	BMI
6	SMA	Number	SMA
7	H-SMA	Number	SMA divided by the height
8	H^2^-SMA	Number	SMA divided by the square of the height
9	W-SMA	Number	SMA divided by the weight
10	Muscle mass	Number	Muscle mass measured by CT
11	Anticancer chemotherapy	2 categories	0) No, 1) Yes
12	Radiation therapy	2 categories	0) No, 1) Yes
13	Comorbidity (hypertension)	2 categories	0) No, 1) Yes
14	Comorbidity (diabetes mellitus)	2 categories	0) No, 1) Yes
15	Date of diagnosis	2 categories	0) No, 1) Yes
16	Date of death	Number	Date (0 being alive)
17	Survival period	Number	Weeks (0 being alive)

ID, identity document; BMI, body mass index; SMA, skeletal muscle area; H, height; W, weight; CT, computed tomography.

We estimated SMA by measuring muscle density at the lumbar skeletal muscle area using computed tomography (CT) with a window range from -29 to 150 Hounsfield Unit (HU). The images were acquired at DIRAMS using a multidetector computed tomography (MDCT) scanner (Brilliance, iCT SP, Philips, USA; X-ray tube voltage: 120 kVp; effective tube current-time product: 120–180 mAs; pitch: 1.173, matrix size: 512 × 512). The thickness of each slice was 5.0 mm. We hypothesized that SMA has a correlation with the survival of patients with stage IV breast cancer and used it as an input for the AI model. In addition, we derived variant features of SMA such as H-SMA, H^2^-SMA and W-SMA as extra inputs for the AI model. H-SMA was calculated as SMA divided by the height; H^2^-SMA was calculated as SMA divided by the square of the height; W-SMA was calculated as SMA divided by the weight. In addition, we investigated the effect of muscle mass, which was measured by CT. For comorbidities, we determined the presence of hypertension (yes or no) and diabetes mellitus (yes or no). Furthermore, we assessed whether the patients received anticancer chemotherapy or/and radiation therapy.

Among the 17 variables, we excluded ID, weight, height, date of diagnosis, date of death, and survival period because the weight and height variables were reflected by H-SMA, H^2^-SMA, and W-SMA. In addition, date of diagnosis, date of death, and survival period variables were used as a label for classifying the patients into the surviving and the deceased groups. Finally, we used 11 variables for our AI model input features, which are age, BMI, muscle mass, anticancer chemotherapy, radiation therapy, SMA, H-SMA, H^2^-SMA, W-SMA, hypertension, and diabetes mellitus. As the AI model output, we classified patients based on whether the patient had survived for 5 years after the date of diagnosis: deceased or survived. Table 2 summarizes the statistics from the surviving group (*n* = 20, 9.52%) and the deceased group (*n* = 190, 90.47%).

**TABLE 2:** Statistical summary of clinical features from the survival group (*n* = 20, 9.52%) and death group (*n* = 190, 90.47%).

No	Data	deceased group (*n* = 190)	survived group (*n* = 20)	*p*
1	Age	54.32 ± 9.76	57.75 ± 6.86	0.124
2	BMI	23.37 ± 3.40	23.55 ± 3.20	0.768
3	Muscle mass	6.14 ± 0.90	6.02 ± 0.68	0.369
4	Anticancer chemotherapy	183/190 (96%)	19/20 (95%)	0.947
5	Radiation therapy	145/190 (76%)	12/20 (60%)	0.077
6	SMA	114.94 ± 16396	120.80 ± 17.70	0.659
7	H-SMA	73.22 ± 10.46	76.24 ± 10.80	0.404
8	H^2^-SMA	46.74 ± 6.85	48.16 ± 6.94	0.459
9	W-SMA	2.01 ± 0.21	2.05 ± 0.22	0.893
10	Hypertension	27/190 (14%)	6/20 (30%)	0.072
11	Diabetes mellitus	19/190 (10%)	0/20 (0%)	0.036

ID, identity document; BMI, body mass index; SMA, skeletal muscle area; H, height; W, weight; CT, computed tomography.

### Data split, data balancing, and cross-validation

In this study, our data consist of training, validation, and testing data. We first split the data of 210 patients into training and testing data at a ratio of 8:2 in a stratified fashion. The testing set was used only for an independent test of our developed AI model and never used for training and internal validation. Table 3 summarizes the training and testing datasets.

**TABLE 3:** Summary of training, validation, and testing datasets.

	Deceased group	Survived group	Total
Training data	152	16	168
Testing data	38	4	42
Total	190	20	210

Using the training data, we performed a 5-fold cross-validation to confirm the generalization ability of the model. The training dataset (n = 168) was first randomly shuffled and divided into five equal groups in a stratified manner. Subsequently, four groups were selected for training the model, and the remaining group was used for internal validation. This process was repeated five times by shifting the internal validation group. Based on the 5-fold cross-validation, we finalized our AI model, which is described in the following sections. We evaluated the performance of our AI model using the isolated testing data.

Here, since the number of deceased patients (90%) was much higher than the number of surviving patients (10%), we up-sampled the surviving patient data by using the Synthetic Minority Over-sampling Technique (SMOTE) with Tomek links (Batista et al., 2004) during the model update. By balancing the numbers in both groups, we attempted to avoid any bias of the model toward the deceased patient data.

### Preprocessing

We performed normalization of the datasets, which is a common requirement for machine learning algorithms (equation (DeSantis et al., 2019)). Normalization reduces the range of each feature between 0 and 1 without changing the normal distribution of original data.

$${Data\;}_{normallized}=\frac{Data-\min\left(train\right)}{\max\left(train\right)-\min\left(train\right)},$$ (1)

where min(train) and max(train) are the minimum and maximum values, respectively, for each feature from the training dataset. Normalization was applied to the training and testing datasets.

### Feature selection

To analyze the effects of 11 features, we first applied the data to six different machine learning algorithms: logistic regression (LR), support vector machine (SVM), K-Nearest Neighbor algorithm (KNN), random forest (RF), adaptive boosting (AdaBoost), and extreme gradient boosting (XGBoost). For XGBoost, we found the optimized parameters: maximum depth of 2, learning rate of 0.1, the number of tree estimators of 50, regularization parameter of 0.8, fraction of observation of 0.2 and fraction of columns of 0.8. For Adaboost, we found the parameters: the number of tree estimators of 50 and learning rate of 0.15. For RF, we found the parameters: the number of tree estimators of 30, maximum depth of 4 and maximum features of 7. For KNN, we found the parameters: the number of neighbors of 2, Minkowski distance of 1, and leaf size of 30. For SVM, we found the parameters: regularization parameter of 1 and radial basis function (RBF). For LR, we found the parameters: inverse of regularization strength of 1 and L2-norm.

We found the best classifier using the 5-fold cross-validation and used 5 evaluation indicators to select the best model. The evaluation indicators include specificity, sensitivity, accuracy, balanced accuracy, and area under receiver operating characteristics (AUROC). In particular, we used balanced accuracy as the main evaluation indicator due to the imbalance between the surviving and deceased patient data. The 5-fold cross-validation provides five sets of evaluation indicators for each classifier. We averaged these values to evaluate the classifier (equation (Miller et al., 2019)).

$$Balanced\;Accuracy=\frac{Sensitivity+Specificity}{2},$$ (2)

After we selected the best classifier, we performed the feature importance analysis, which lists the features in the order of importance. To develop the final AI model, we used a deep neural network (DNN). For the input layer, we ranked the features according to their importance from the best classifier and increased the number of used top features in the input layer from 1 to 11.

### AI prediction model development

To develop the final DNN-based AI model for survival prediction, we searched for hyperparameters such as layer depth and width. We investigated up to 5 hidden layers and each layer depth (node) up to the previous layer depth (node). We applied batch normalization for the fully connected (FC) layers as hidden layers. The last FC layer was fed into a sigmoid layer, which is an output layer providing the probability for five-year survival. We trained the models with an ADAM optimizer and binary cross-entropy cost function with a learning rate of 0.0001 and a batch size of 3. We implemented the models using python (version: 3.9) with TensorFlow (version: 2.8.0), Keras (version: 2.8.0), NumPy (version: 1.20.3), Pandas (version: 1.3.4), Matplotlib (version: 3.4.3), and Scikit-learn (version: 1.0.2).

**FIGURE 1:**
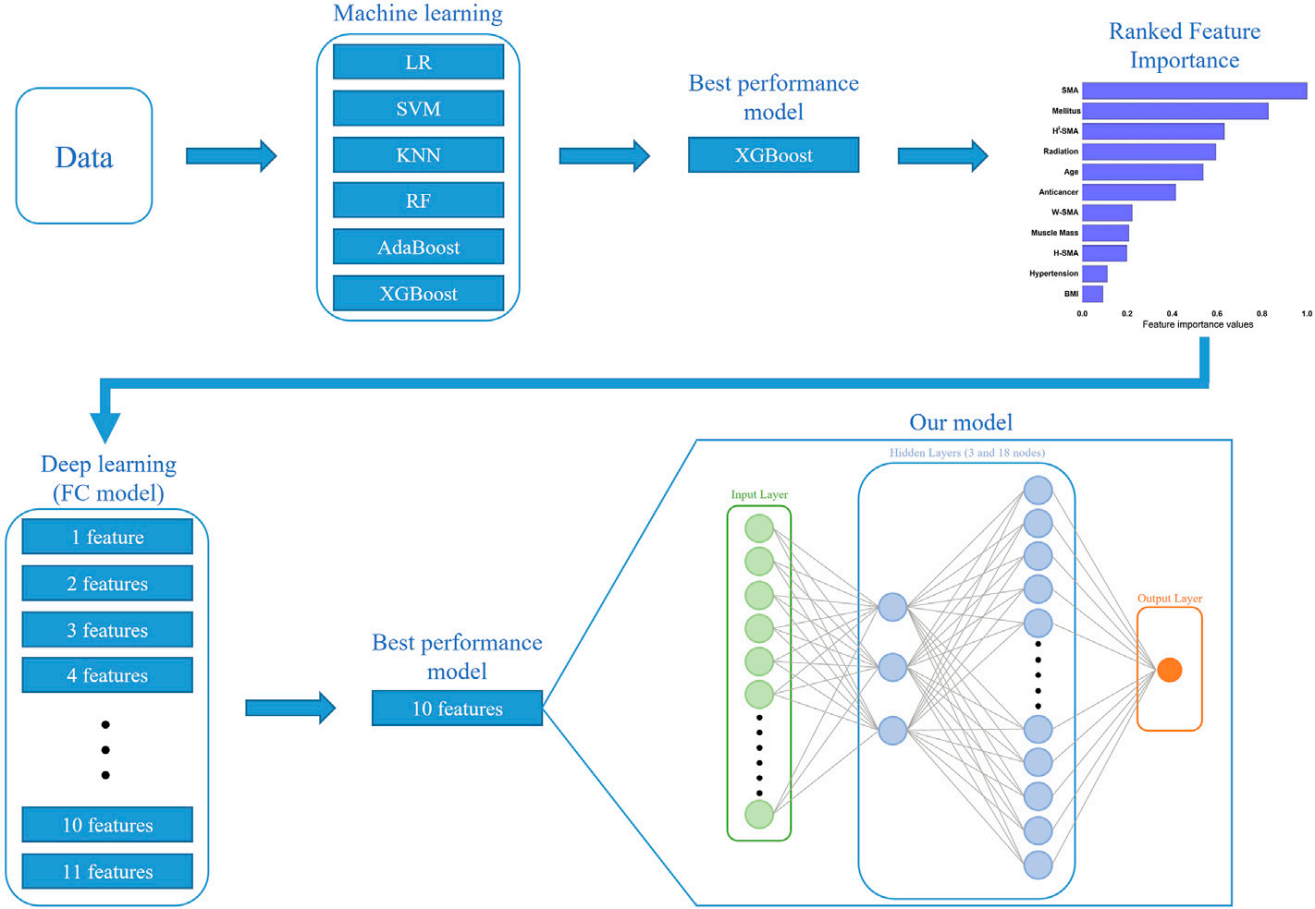
Overall strategy and architecture to build our proposed 4-layer DNN model: given the cross-validation accuracy analysis from the best classifier, XGBoost, we modeled with a 4-layer DNN using the top 10 features providing the best cross-validation accuracy. Results of feature importance analysis from XGBoost: SMA is with the highest importance.

For each set of top features, we found the best cross-validation accuracy using the two metrics of AUROC and balanced accuracy. Given the cross-validation accuracy analysis, we finally modeled with a 4-layer DNN using the top 10 features providing the best cross-validation accuracy. The 4-layer DNN comprised an input layer, two FC layers as hidden layers, and an output layer. The input layer was fed into a series of two FC layers consisting of 3 and 18 nodes, respectively. Figure 1 illustrates overall strategy and architecture to build our proposed 4-layer DNN model. Finally, the prediction performance of our proposed 4-layer DNN model was evaluated with the isolated testing dataset (*n* = 42).

### Public website deployment

We deployed our model on a public web server. After accessing the website, a user enters the patient information of age, BMI, muscle mass, anticancer chemotherapy, radiation therapy, SMA, H-SMA, H^2^-SMA, W-SMA, hypertension, and diabetes mellitus, which are encoded to the website server. The user can immediately obtain the prediction result of their five-year survival of stage IV breast cancer.

## Results

### Ranked feature importance

Table 4 summarizes the Cross-validation results from six different machine learning algorithms of LR, SVM, KNN, RF, Adaboost, and XGBoost. The results show that the XGBoost model is the best classifier to predict five-year survival in stage IV breast cancer patients. The XGBoost model has an accuracy value of 0.83, balanced accuracy of 0.66, and AUROC of 0.69, which are higher than the corresponding values obtained from other algorithms.

**TABLE 4:** Cross-validation results from six different machine learning algorithms (mean ± standard deviation).

Cross-validation results					
Model	Sensitivity	Specificity	Accuracy	Balanced accuracy	AUROC
LR	0.29 ± 0.26	0.62 ± 0.10	0.59 ± 0.08	0.46 ± 0.12	0.51 ± 0.15
SVM	0.45 ± 0.34	0.72 ± 0.13	0.69 ± 0.11	0.58 ± 0.16	0.52 ± 0.21
KNN	0.53 ± 0.33	0.61 ± 0.10	0.60 ± 0.08	0.57 ± 0.15	0.56 ± 0.20
RF	0.40 ± 0.38	0.71 ± 0.05	0.67 ± 0.04	0.55 ± 0.18	0.53 ± 0.20
AdaBoost	0.31 ± 0.21	0.83 ± 0.02	0.78 ± 0.04	0.57 ± 0.11	0.56 ± 0.08
XGBoost	0.45 ± 0.17	0.88 ± 0.07	0.83 ± 0.07	0.66 ± 0.10	0.69 ± 0.16

LR, logistic regression; SVM, support vector machine; KNN, K-Nearest Neighbor algorithm; RF, random forest; AdaBoost, adaptive boosting; XGBoost, extreme gradient boosting.

**FIGURE 2:**
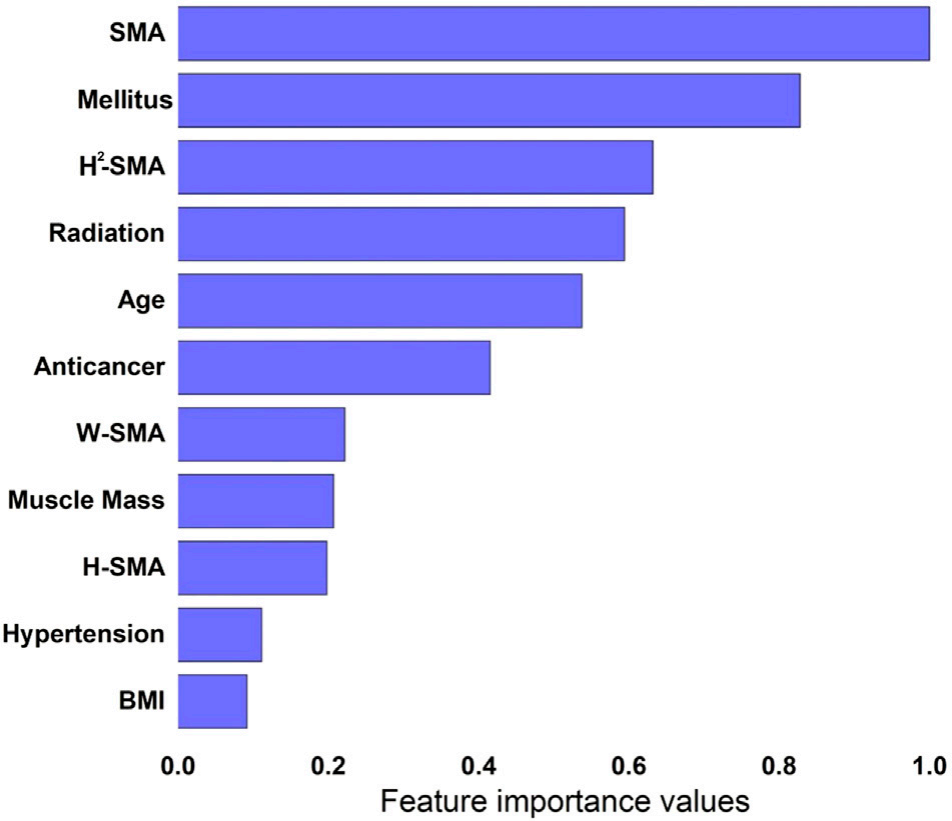
Results of feature importance analysis from XGBoost: SMA is with the highest importance value, followed by diabetes mellitus, H^2^-SMA, radiation therapy, age, anticancer chemotherapy, W-SMA, muscle mass, H-SMA, hypertension, and BMI.

Next, we performed feature importance analysis using the XGBoost model. Figure 2 shows the results of the ranked feature importance from the XGBoost model, which indicate that SMA had the highest importance value, followed by diabetes mellitus, H^2^-SMA, radiation therapy, age, anticancer chemotherapy, W-SMA, muscle mass, H-SMA, hypertension, and BMI in that order.

### K-fold cross-validation results of our AI model

**FIGURE 3:**
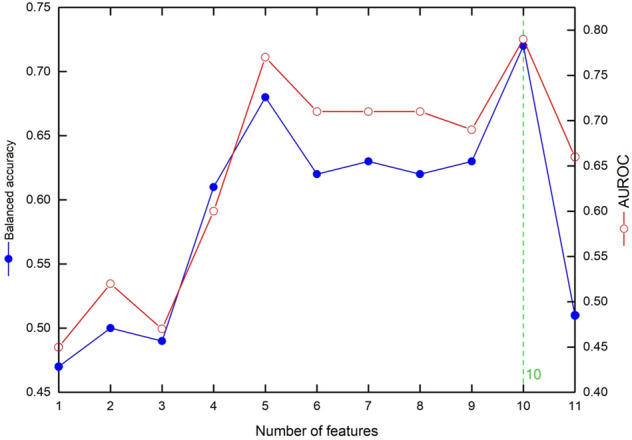
The influence of the ranked features on cross-validation accuracy metrics: the values of balanced accuracy and AUROC according to the number of top features.

We used DNN to investigate the cross-validation performance with AUROC and balanced accuracy. Figure 3 shows the influence of the ranked features. We investigated the values of AUROC and balanced accuracy according to the number of top features. The results show that both AUROC and balanced accuracy reach the highest values when the top 10 features are used for the input layer. Therefore, we selected the top 10 features in our AI model, which yielded a sensitivity of 64%, specificity of 79%, accuracy of 77%, balanced accuracy of 72%, and AUROC of 0.79. Table 5 summarizes the cross-validation results from DNN according to the number of top features. Moreover, note that the DNN provides higher accuracy than XGBoost.

**TABLE 5:** Cross-validation results from DNN according to the number of top features (mean ± standard deviation).

Cross-validation results (*n* = 34)					
The number of features	Sensitivity	Specificity	Accuracy	Balanced accuracy	AUROC
1	0.73 ± 0.24	0.22 ± 0.09	0.27 ± 0.06	0.47 ± 0.09	0.45 ± 0.15
2	0.73 ± 0.32	0.27 ± 0.10	0.32 ± 0.05	0.50 ± 0.11	0.52 ± 0.13
3	0.53 ± 0.40	0.45 ± 0.06	0.46 ± 0.03	0.49 ± 0.17	0.47 ± 0.16
4	0.66 ± 0.36	0.56 ± 0.08	0.57 ± 0.06	0.61 ± 0.17	0.60 ± 0.19
5	0.59 ± 0.24	0.76 ± 0.11	0.75 ± 0.08	0.68 ± 0.08	0.77 ± 0.09
6	0.53 ± 0.33	0.72 ± 0.07	0.70 ± 0.06	0.62 ± 0.15	0.71 ± 0.16
7	0.46 ± 0.33	0.83 ± 0.10	0.78 ± 0.08	0.63 ± 0.15	0.71 ± 0.20
8	0.49 ± 0.14	0.74 ± 0.10	0.72 ± 0.09	0.62 ± 0.09	0.71 ± 0.15
9	0.49 ± 0.13	0.77 ± 0.07	0.74 ± 0.09	0.63 ± 0.05	0.69 ± 0.09
10	0.64 ± 0.31	0.79 ± 0.09	0.77 ± 0.07	0.72 ± 0.13	0.76 ± 0.12
11	0.24 ± 0.24	0.78 ± 0.05	0.73 ± 0.04	0.51 ± 0.10	0.66 ± 0.14

### Testing data results

With the isolated testing dataset (*n* = 42), our proposed 4-layer DNN shows a sensitivity of 75.00%, specificity of 78.94%, accuracy of 78.57%, balanced accuracy of 76.97%, and AUROC of 0.90, as summarized in Table 6. With respect to balanced accuracy, our 4-layer DNN provided the highest value, followed by XGBoost and AdaBoost. With respect to the AUROC, our 4-layer DNN also provided the highest value, followed by XGBoost and KNN.

**TABLE 6:** Testing data results.

Model	TP	TN	FP	FN	Sensitivity	Specificity	Accuracy	Balanced accuracy	AUROC
4-layer DNN (Proposed)	3	30	8	1	0.75	0.7894	0.7857	0.7697	0.90
LR	2	21	17	2	0.5	0.4473	0.4523	0.4736	0.47
SVM	2	24	14	2	0.5	0.6315	0.6190	0.5657	0.55
KNN	2	37	7	2	0.5	0.8157	0.7857	0.6578	0.65
RF	2	27	11	2	0.5	0.7105	0.6904	0.6052	0.55
AdaBoost	1	33	5	3	0.25	0.8684	0.8095	0.5592	0.58
XGBoost	2	33	5	2	0.5	0.8684	0.8139	0.6842	0.67

TP, true positives; TN, true negatives; FP, false positives; FN, false negatives; AUROC, area under receiver operating characteristics; DNN, deep neural network; DT, decision tree; SVM, support vector machine; KNN, K-Nearest Neighbor algorithm; RF, random forest; AdaBoost, adaptive boosting; XGBoost, extreme gradient boosting.

### Web application deployment

**FIGURE 4:**
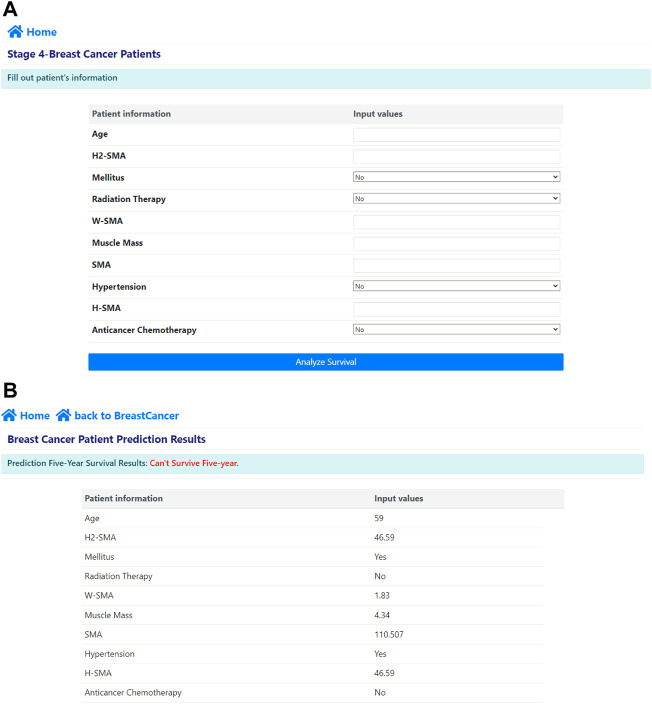
Our deployed web application: **(A)** input windows where the user inputs the patient’s data and **(B)** the predicted five-year survival results after entering the patient’s data.

We deployed our AI on a public website (http://ai-wm.khu.ac.kr/BreastCancer/) for predicting five-year survival on the website using patients’ data. Figure 4 shows the predictions provided by the web application. In the application, a user can input the patient information including age, BMI, muscle mass, anticancer chemotherapy, radiation therapy, SMA, H-SMA, H^2^-SMA, W-SMA, the presence of hypertension or diabetes mellitus as shown in Figure 4A. After the user enters the patient information, he or she can immediately obtain the prediction probability result of five-year survival of stage IV breast cancer as shown in Figure 4B.

## Discussion and conclusion

We presented an AI model that can predict five-year survival in metastatic breast cancers using 11 input variables, including age, BMI, SMA, H-SMI, H^2^-SMA, W-SMA, muscle mass, anticancer chemotherapy, radiation therapy, and comorbidities (hypertension and diabetes mellitus). We adopted a 4-layer deep neural network, which can provide high sensitivity (75.00%), specificity (78.94%), accuracy (78.57%), balanced accuracy (76.97%), and an AUROC of 0.90.

Interestingly, in our study, the ranked feature importance analysis demonstrated that SMA had the highest importance value, followed by diabetes mellitus, H^2^-SMA, radiation therapy, age, anticancer chemotherapy, W-SMA, muscle mass, H-SMA, hypertension, and BMI. This result indicated that sarcopenia has great prognostic value for predicting five-year survival, which is consistent with many previous findings emphasizing the relationship between sarcopenia and survival (Villasenor et al., 2012; Shachar et al., 2017; Caan et al., 2018; Song et al., 2018; Weinberg et al., 2018).

Indeed, a special program consisting of diet and exercise training has been adopted to ameliorate sarcopenia and improve the general health status for breast cancer patients (Demark-Wahnefried et al., 2008; Bowen et al., 2015). Our AI application could help cancer patients know their current mortality risk, motivating them and their doctors to improve the patients’ health by diet and exercise training to gain muscle. In future work, we also plan to organize the training program for appropriate diet and exercise and track the prognostic outcomes for patients with stage IV breast cancer. Since our AI model emphasized SMA as the highest prognostic factor, we will focus on developing programs that can effectively increase skeletal muscle mass and function. There are many effective cancer treatments such as targeted agents, hormonal agents, and immunotherapeutic agents. These treatments can be continued as long as patients can tolerate them, which largely depends on skeletal muscle mass and function (DeSantis et al., 2014). Skeletal muscle plays an important role in disease resistance and the endurance of chemotherapy (Demark-Wahnefried et al., 2008; Lee et al., 2019). Patients with sarcopenia tend to have severe chemotherapy-related side effects, hospitalizations, and treatment interruptions, all of which influence the overall survival (Prado et al., 2009). Therefore, the concept of skeletal muscle gain for survival gain is being revolutionized.

Muscle is known to have metabolic and endocrine functions (Kaji, 2016). For example, myokines released from muscle are closely related to the molecules known to increase glucose production in the liver, lipolysis in adipose tissue, pancreatic beta-cell viability, and insulin secretion (Batista et al., 2004; Lee et al., 2019). Indeed, many studies have demonstrated a close relationship between sarcopenia and diabetes mellitus. Of note, in our study, diabetes mellitus was the second highest prognostic factor in the ranked feature importance analysis, which also underlines the important relationship between sarcopenia and diabetes mellitus. Therefore, the training program we envision will also consider a personalized diet that can prevent or effectively manage diabetes.

In this study, we have proposed a 4-layer deep neural network based on the top 10 ranked features. The accuracy of our AI model is high enough to be used in clinical practice (AUROC of 0.90). To find the best model for predicting five-year survival in patients with stage IV metastatic breast cancer, we first analyzed the feature importance using XGBoost, which provided the best accuracy among several machine learning models, and extended the results to DNN for our final AI model. Finally, the model outperformed any machine learning algorithms including XGBoost.

A few researchers have built a prognostic AI model in breast cancer patients (Abdikenov et al., 2019; Simsek et al., 2020; Haque et al., 2022; Lin et al., 2022). These AI models have utilized limited clinical data without any body composition measures. More specifically, in (Abdikenov et al., 2019; Simsek et al., 2020; Haque et al., 2022), AI models were developed based on the public dataset named the Surveillance, Epidemiology and End Results (SEER). The dataset includes includes the information of age, T stage, N stage, tumor size, estrogen status, progesterone status, regional lymph nodes and survival months. In (Lin et al., 2022), the tumor node metastasis (TNM) stage, BMI, regional lymph nodes, surgery and tumor size/characteristics were considered. Our study is the first to build an AI prediction model considering body composition measures. One of the main contributions is that we collected the data from the patients with stage IV metastatic breast cancer only and developed the AI model to predict the mortality. Moreover, for the first time, we could successfully deploy the AI model in a public website for only the patients with stage IV metastatic breast cancer, whose survival rate is very low.

There are several limitations in our study. First, this study was performed in only one center, which may reduce the generalizability of our results. However, our hospital is a third referral center and actively receives patients from many community centers. Second, our model lacks many other clinical variables such as tumor genetic profiling, as well as details about chemotherapy agents. However, these tumor factors and treatment factors vary over time. Therefore, we focused on host factors that can be improved by human efforts and hope that our AI web application helps better management of the therapy of stage IV breast cancer patients.

In conclusion, we successfully deployed an AI model that can predict five-year survival in patients with stage IV metastatic breast cancer, mainly based on host factors and sarcopenia. Its web application allows easy access to the AI model and predicts five-year survival with a small number of input variables. We expect that this application will help patients become aware of the importance of host factors, including sarcopenia, for long-term survival in metastatic breast cancers and eventually help in achieving survival gains in these patients.

## Data Availability

The original contributions presented in the study are included in the article/supplementary material, further inquiries can be directed to the corresponding authors.

## References

[B1] Abdikenov B. Iklassov Z. Sharipov A. Hussain S. Jamwal P. K. (2019). Analytics of heterogeneous breast cancer data using neuroevolution. IEEE Access 7, 18050–18060. 10.1109/access.2019.2897078

[B2] Batista G. E. Prati R. C. Monard M. C. (2004). A study of the behavior of several methods for balancing machine learning training data. SIGKDD Explor. Newsl. 6 (1), 20–29. 10.1145/1007730.1007735

[B3] Bonotto M. Gerratana L. Poletto E. Driol P. Giangreco M. Russo S. (2014). Measures of outcome in metastatic breast cancer: Insights from a real-world scenario. Oncologist 19 (6), 608–615. 10.1634/theoncologist.2014-0002 24794159 PMC4041678

[B4] Bowen T. S. Schuler G. Adams V. (2015). Skeletal muscle wasting in cachexia and sarcopenia: Molecular pathophysiology and impact of exercise training. J. Cachexia Sarcopenia Muscle 6 (3), 197–207. 10.1002/jcsm.12043 26401465 PMC4575550

[B5] Caan B. J. Cespedes Feliciano E. M. Prado C. M. Alexeeff S. Kroenke C. H. Bradshaw P. (2018). Association of muscle and adiposity measured by computed tomography with survival in patients with nonmetastatic breast 1cancer. JAMA Oncol. 4 (6), 798–804. 10.1001/jamaoncol.2018.0137 29621380 PMC6584322

[B6] Demark-Wahnefried W. Case L. D. Blackwell K. Marcom P. K. Kraus W. Aziz N. (2008). Results of a diet/exercise feasibility trial to prevent adverse body composition change in breast cancer patients on adjuvant chemotherapy. Clin. Breast Cancer 8 (1), 70–79. 10.3816/CBC.2008.n.005 18501061

[B7] DeSantis C. Ma J. Bryan L. Jemal A. (2014). Breast cancer statistics, 2013. Ca. Cancer J. Clin. 64 (1), 52–62. 10.3322/caac.21203 24114568

[B8] DeSantis C. E. Ma J. Gaudet M. M. Newman L. A. Miller K. D. Goding Sauer A. (2019). Breast cancer statistics, 2019. Ca. Cancer J. Clin. 69 (6), 438–451. 10.3322/caac.21583 31577379

[B9] Gobbini E. Ezzalfani M. Dieras V. Bachelot T. Brain E. Debled M. (2018). Time trends of overall survival among metastatic breast cancer patients in the real-life esme cohort. Eur. J. Cancer 96, 17–24. 10.1016/j.ejca.2018.03.015 29660596

[B10] Haque M. N. Tazin T. Khan M. M. Faisal S. Ibraheem S. M. Algethami H. (2022). Predicting characteristics associated with breast cancer survival using multiple machine learning approaches. Comput. Math. Methods Med. 2022, 1249692. Epub 2022/05/06. 10.1155/2022/1249692 35509861 PMC9060999

[B11] Ho-Huynh A. Tran A. Bray G. Abbot S. Elston T. Gunnarsson R. (2019). Factors influencing breast cancer outcomes in Australia: A systematic review. Eur. J. Cancer Care 28 (4), e13038. 10.1111/ecc.13038 30919536

[B12] Kaji H. (2016). Effects of myokines on bone. Bonekey Rep. 5, 826. Epub 2016/09/01. 10.1038/bonekey.2016.48 27579164 PMC4954587

[B13] Lee K. Shin Y. Huh J. Sung Y. S. Lee I. S. Yoon K. H. (2019). Recent issues on body composition imaging for sarcopenia evaluation. Korean J. Radiol. 20 (2), 205–217. 10.3348/kjr.2018.0479 30672160 PMC6342757

[B14] Lin C. Y. Chien T. W. Chen Y. H. Lee Y. L. Su S. B. (2022). An app to classify a 5-year survival in patients with breast cancer using the convolutional neural networks (cnn) in microsoft excel: Development and usability study. Med. Baltim. 101 (4), e28697. Epub 2022/01/29. 10.1097/MD.0000000000028697 PMC8797502 35089226

[B15] Miller K. D. Nogueira L. Mariotto A. B. Rowland J. H. Yabroff K. R. Alfano C. M. (2019). Cancer treatment and survivorship statistics, 2019. Ca. Cancer J. Clin. 69 (5), 363–385. 10.3322/caac.21565 31184787

[B16] Prado C. M. M. Baracos V. E. McCargar L. J. Reiman T. Mourtzakis M. Tonkin K. (2009). Sarcopenia as a determinant of chemotherapy toxicity and time to tumor progression in metastatic breast cancer patients receiving capecitabine treatment. Clin. Cancer Res. 15 (8), 2920–2926. 10.1158/1078-0432.ccr-08-2242 19351764

[B17] Shachar S. S. Deal A. M. Weinberg M. Nyrop K. A. Williams G. R. Nishijima T. F. (2017). Skeletal muscle measures as predictors of toxicity, hospitalization, and survival in patients with metastatic breast cancer receiving taxane-based chemotherapy. Clin. Cancer Res. 23 (3), 658–665. 10.1158/1078-0432.CCR-16-0940 27489287 PMC5290138

[B18] Simsek S. Kursuncu U. Kibis E. AnisAbdellatif M. Dag A. (2020). A hybrid data mining approach for identifying the temporal effects of variables associated with breast cancer survival. Expert Syst. Appl. 139, 112863. 10.1016/j.eswa.2019.112863

[B19] Song E. J. Lee C. W. Jung S. Y. Kim B. N. Lee K. S. Lee S. (2018). Prognostic impact of skeletal muscle volume derived from cross-sectional computed tomography images in breast cancer. Breast Cancer Res. Treat. 172 (2), 425–436. 10.1007/s10549-018-4915-7 30132218

[B20] Villasenor A. Ballard-Barbash R. Baumgartner K. Baumgartner R. Bernstein L. McTiernan A. (2012). Prevalence and prognostic effect of sarcopenia in breast cancer survivors: The heal study. J. Cancer Surviv. 6 (4), 398–406. 10.1007/s11764-012-0234-x 23054848 PMC3747827

[B21] Weinberg M. S. Shachar S. S. Muss H. B. Deal A. M. Popuri K. Yu H. (2018). Beyond sarcopenia: Characterization and integration of skeletal muscle quantity and radiodensity in a curable breast cancer population. Breast J. 24 (3), 278–284. 10.1111/tbj.12952 29139618 PMC6414810

